# Biological Activity Evaluation and In Silico Studies of Polyprenylated Benzophenones from *Garcinia celebica*

**DOI:** 10.3390/biomedicines9111654

**Published:** 2021-11-10

**Authors:** Yenni Pintauli Pasaribu, Arif Fadlan, Sri Fatmawati, Taslim Ersam

**Affiliations:** 1 Department of Chemistry, Faculty of Science and Data Analytics, Institut Teknologi Sepuluh Nopember (ITS), Kampus ITS Sukolilo, Surabaya 60111, Indonesia; pasaribu@unmus.ac.id (Y.P.P.); afadlan@chem.its.ac.id (A.F.); fatma@chem.its.ac.id (S.F.); 2 Department of Chemistry Education, Faculty of Teacher Training and Education, Musamus University, Merauke 99600, Indonesia

**Keywords:** *Garcinia celebica*, polyprenylated benzophenone, antioxidant, cytotoxicity, antiplasmodial, molecular docking

## Abstract

This study aimed to isolate polyprenylated benzophenones from the rootbark of *Garcinia celebica* and assess their activities in vitro and in silico. The antioxidant activity was evaluated by the DPPH, ABTS, and FRAP methods. The cytotoxicity was evaluated against HeLa, MCF-7, A549, and B16 cancer cell lines. The antiplasmodial activity was performed against the chloroquine-sensitive *Plasmodium falciparum* strain 3D7. Molecular docking was analyzed on alpha-estrogen receptor (3ERT) and *P. falciparum* lactate dehydrogenase enzyme (1CET). The prediction of ADMET for the compounds was also studied. For the first time, (-)-cycloxanthochymol, isoxanthochymol, and xanthochymol were isolated from the root bark of *Garcinia celebica*. The antioxidant and cytotoxicity evaluation showed that all benzophenones exhibited antioxidant activity compared to gallic acid and quercetin as positive controls and also exhibited strong activity against HeLa, MCF-7, A549, and B16 cell lines compared to cisplatin as the positive control. The antiplasmodial evaluation showed that isoxanthochymol exhibited activity against the chloroquine-sensitive *P. falciparum* strain 3D7. In addition, the in silico molecular docking study supported in vitro activities. The ADMET analysis also indicated the isolated benzophenones are potential oral drug candidates.

## 1. Introduction

Cancer (or neoplasm and malignant tumor) is defined as an abnormal growth of cells beyond normal boundaries, to breach adjacent parts of the body and spread out to other organs, causing metastasis. This large group of disease is one of the leading causes of death in the world, with about 9.6 million deaths recorded in 2018 alone [1]. In Indonesia, breast cancer has the highest incidence rate of 42.1 per 100,000 population with an average death rate of 17 from the same population [2]. Meanwhile, malaria is considered a major global health problem and affects a large population of the world, especially in low-income countries, with about 229 million cases and 409 deaths recorded in 2019 alone. Malaria is caused by *Plasmodium* parasites transferred to humans through the bites of infected female *Anopheles* mosquitoes. According to The Ministry of Health of The Republic of Indonesia, *P. falciparum* is the prevalent parasite causing the endemic malaria in Papua, East Indonesia [3]. Furthermore, medicinal plants have been used worldwide as complementary or alternative medicines; therefore, studies on these medicinal plants, including biological and pharmacological evaluations, are important for drug discovery. Numerous medicinal compounds have been discovered from medicinal plants, for instance, the cancer drugs vincristine and vinblastine were isolated from *Catharanthus roseus*, *paclitaxel* was isolated from *Taxus baccata*, the malaria drug quinine was obtained from Cinchona *ledgeriana*, and artemisinin was isolated for the first time from *Artemisia annua* L. [4,5].

The *Garcinia* genera (family Clusiaceae) contains numerous plants rich in compounds with interesting biological properties, including antioxidant, anticancer, and antimalaria properties, for instance, xanthones, and polyprenylated benzophenones. Xanthones vieillardixanthone, isocudraniaxanthone A, 1,3,7-trihydroxyxanthone, cheffouxanthone, smeathxanthone, and benzophenones 2,4,6,3′,4′,6′-hexahydroxybenzophenone and garcinol exhibited impressive antioxidant activity against DPPH [6,7,8,9,10,11]. In addition, xanthones 9-hydroxycalabaxanthone, macluraxanthone, garcinoxanthocins A and B, 14-deoxygarcinol garcicowin C, isogarcinol, gaudichaudione H, cantleyanone A, oliganthin I, garcibractatin A, forbesione, isoforbesione, gambogic acid [12,13,14,15,16,17,18], and polyprenylated benzophenones guttiferone E, guttiferone H, garcinol, and picrorhizone H [9,19,20] were shown to exhibit cytotoxicity against cancer cell lines. Mckeanianones A–C, bannaxanthones E and I, pancixanthones A and B, and assiguxanthone A have also been reported to be active against *P. falciparum* strain TM4 and K1; α-mangostin and β-mangostin have been reported active against *P. falciparum* strain D6 and W2; while subelliptenone and 12b-hydroxy-des-D-garcigerrin A have also been reported to be active against *P. falciparum* strain 3D7 [21,22,23]. Meanwhile, several polyprenylated benzophenones, including guttiferone E and H, were discovered to be active against *P. falciparum* strain D6 and W2 [22].

*Garcinia celebica* L. is a medium-to-large-sized tree, which grows up to 30 m in height, with yellow latex and sour but edible fruit. *G. celebica* L. has been empirically used as a folk medicine to treat endemic malaria and breast tumor medication by local people in Papua. However, only a few phytochemical and biological studies on *G. celebica* have been published. Previous phytochemical studies on this species have isolated triterpenoids friedelin, garcihombronane D, methyl-3α,23-dihydroxy-17,14-friedolanstan-8,14,24-trien-26-oat, 3,23-dioxo-9,16-lanostadien-26-oic acid, (*E*)-3β,9α-dihydroxylanosta-24-en-26-oic acid, (22*Z*,24*E*)-3-oxo-17,14-friedolanosta-8,14,22,24-tetraen-26-oic acid, (24*E*)-3-oxo-17,14-friedolanosta-8,14,24-trien-26-oic acid, (22*Z*,24*E*)-9α-hydroxy-3-oxo-17,13-friedolanosta-12,22,24-trien-26-oic acid, mangiferolic acid, (22*Z*,24*E*)-9α-hydroxy-3-oxo-17,14-friedolanosta-14,22,24-trien-26-oic acid, (22*Z*,24*E*)-9α-hydroxy-3-oxo-13α,30-cyclo-17,13-friedolanosta-22,24-dien-26-oic acid, (22*Z*,24*E*)-3β-acetoxy-9α,hydroxy-17,14-friedolanosta-14,22,24-trien-26-oic acid, and (24E)-3b-acetoxy-9α-hydroxy-17,14-friedolanosta-14,24-dien-26-oic acid, xanthones macluraxanthone, toxyloxanthone B, nigrolineaxanthone A, nigrolineaxanthone E, 6-deoxyjacareubin, 6-deoxyisojacareubin, morusignin A, isocudraniaxanthone B, and depsidone garcinisidone H. Several of these compounds exhibited antiplasmodial and anticancer activity [13,24,25]. However, there are no known reports on polyprenylated benzophenones present in *G. celebica*. This study, therefore, aimed to perform isolation and structural elucidation of the isolated compounds, as well as an evaluation of the antioxidant activity, in vitro cytotoxicity against four cancer cell lines (HeLa, MCF-7, A549, and B16), and in vitro antiplasmodial activity against chloroquine-sensitive *P. falciparum* 3D7 strain. In addition, this study provides an explanation of the in silico studies of molecular docking, as well as the ADMET analysis of the compounds. The aims of this study were also in line with the policy of the government of Republic of Indonesia in the health sector, which craves independency in drugs and medicine-related areas. This program encourages researchers to explore bioactive compounds from native medicinal plants of Indonesia to overcome endemic and deadly diseases, such as malaria and cancer.

## 2. Materials and Methods

### 2.1. General Experimental Procedures

**Figure 1:**
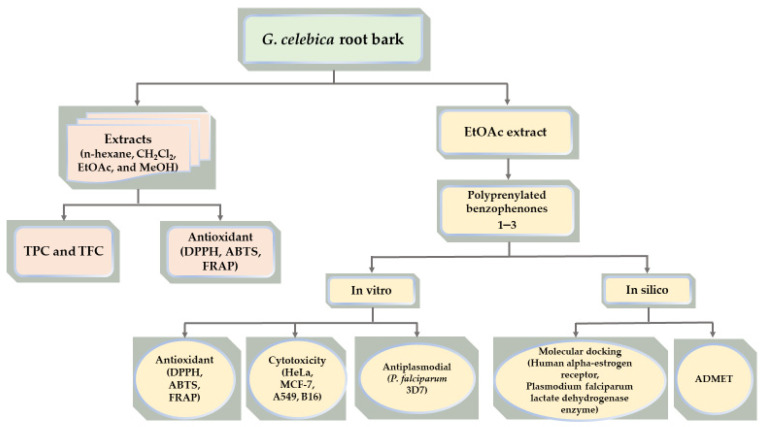
The scheme of the general experimental procedures.

Figure 1 shows the general experimental procedures of this study.

### 2.2. Plant Material

Garcinia celebica root barks were collected from Wasur National Park, Merauke Papua, Indonesia (8°31′16.32″ S, 140°33′26.47″ E). Subsequently, the plant was identified by Mr. Deni Sahroni, and a voucher specimen of this plant was deposited at the Herbarium Bogoriense, Bogor Botanical Garden, Indonesia (specimen number Ch. Koster BW 15509).

### 2.3. Preparation of Root Bark Extracts

The root bark of *G. celebica* (20 kg) was collected, cut into small pieces, sun-dried, and ground into powder using a grinder. Subsequently, 10 g of the powder were extracted by maceration using *n*-hexane, CH_2_Cl_2_, EtOAc, and MeOH (1:10, *w*/*v*) [26], separately, for 24 h at room temperature. The *n*-hexane, CH_2_Cl_2_, EtOAc, and MeOH organic solvents were chosen for the extraction procedure because of their ability to extract the active compounds, affordability, and quick separation from the extract [27]. The extracts were then filtered and concentrated using a rotary evaporator to obtain *n*-hexane, CH_2_Cl_2,_ EtOAc, and MeOH crude extracts.

### 2.4. Extraction and Isolation

The compounds were isolated through vacuum liquid chromatography (VLC) (silica gel 60 G), Sephadex LH-20 (25–100 μm; GE Chemical Corporation), and TLC analysis (silica gel 60 GF_254_, 0.25 mm Merck). The powdered root bark of *G. celebica* (7.5 kg) was extracted with EtOAc (3 days × 10 L) through maceration, and the extract was concentrated in vacuo to yield 966.0 g of extract. The maceration process is a prevalent technique for the extraction of active compounds from plant materials [5]. Generally, the isolation of natural products needs a large amount of sample and maceration is suitable for this condition. Additionally, this method is convenient and very suitable for thermolabile plant materials. Subsequently, 90 g of the extract were fractionated through VLC on silica gel (250.0 g) using 100% *v/v n*-hexane, 100% *v/v* methylene chloride (CH_2_Cl_2_), 100% *v/v* ethyl acetate (EtOAc), and 100% *v/v* methanol (MeOH) to obtain seven fractions (A to F). Fraction E (17 g) was further subjected to VLC on silica gel (150 g) using an *n*-hexane:EtOAc gradient (100:0, 90:10, 80:20, 70:30, 0:100 *v/v*, each 300 mL) to obtain subfractions E1 to E3. Subsequently, compound **1** (327.4 mg) was obtained by recrystallization of subfraction E3 from *n*-hexane. Meanwhile, subfraction E2 (4.08 g) was chromatographed into silica (50 g) using a gradient of *n*-hexane: EtOAc (100:0, 90:10, 80:20, 70:30, 0:100 *v/v*, each 200 mL) to produce 5 subfractions (E2.1 to E2.5). In addition, subfraction E2.3 (2.47 g) was separated on a Sephadex column using CH_2_Cl_2_:MeOH (1:1 *v/v*) to yield 6 subfractions (E2.3.1 to E2.3.6). Subfraction E2.3.3 was then recrystallized from petroleum benzene to obtain compound **2** (56 mg). Additionally, fraction D (37 g) was subjected to VLC on silica gel (250 g) using an *n*-hexane: EtOAc gradient (100:0, 99:1, 98:2, 97:3, 95:5, 94:6, 90:10, 80:20, 70:30, 0:100 *v/v*, each 300 mL) to obtain 7 subfractions (D1 to D7). Subfraction D4 (15 g) was then separated by the same technique using an *n*-hexane:EtOAc gradient (100:0, 95:5, 90:10, 85:15, 0:100 *v/v*, each 200 mL) to yield 8 subfractions (D4.1 to D4.8). Subfraction D4.4 (4.78 g) was further purified through Sephadex using CH_2_Cl_2:_MeOH (1:1 *v/v*) to produce 3 fractions (D4.4.1 to D4.4.3). Subsequently, compound 3 (142 mg) was obtained from subfraction D4.4.2 through recrystallization from *n*-hexane. Subsequently, the compounds were subjected to melting point and UV-Vis spectroscopic evaluations using a micro-melting point apparatus Fischer John (Philip Haris, Calgary, Canada) and a UV-Vis spectrophotometer (Genesys, Thermo Fisher Scientific, Madison, WI, USA), respectively. Meanwhile, NMR characterization (^1HhH^H 500 MHz and ^13^C 125 MHz) was performed using an NMR spectrometer (Agilent, Santa Clara, CA, USA) with DD2 console system with methanol-*d*_4_ (CD_3_OD).

### 2.5. Total Phenolic and Flavonoid Contents

#### 2.5.1. Total Phenolic Content (TPC)

The total phenolic content (TPC) of the *n*-hexane, CH_2_Cl_2_, EtOAc, and MeOH extracts of *G. celebica* was evaluated using Follin–Ciocalteu’s method [26] with minor modifications. For this analysis, 0.5 mL of each extract in MeOH was mixed with 10% Follin–Ciocalteu’s reagent from Sigma-Aldrich (St. Louis, MO, USA) and 2.0 mL of 7.5% aqueous sodium carbonate (Na_2_CO_3_) solution. The mixture was then incubated at 40 °C for 1 h and the absorbance was measured using a UV-Vis spectrophotometer at 765 nm. Subsequently, a standard curve of gallic acid (Sigma-Aldrich) was prepared at a concentration range of 0 to 200 mg/L, and the extracts’ TPCs were calculated as mg GAE (gallic acid equivalent)/g of dry extract.

#### 2.5.2. Total Flavonoid Content (TFC)

The total flavonoid content (TFC) of the *n*-hexane, CH_2_Cl_2_, EtOAc, and MeOH extracts of *G. celebica* was measured spectrophotometrically, using the reported method [28], with minor modification. For this evaluation, 0.5 mL of each extract (100–1000 ppm) were mixed with 0.5 mL of 2% AlCl_3_ (Sigma-Aldrich) solution in MeOH, incubated for 1 h, and subjected to spectroscopic evaluation at 415 nm. Subsequently, a standard curve of quercetin (Sigma-Aldrich) was prepared at a concentration range of 0 to 50 mg/L, and the extracts’ TFCs were expressed as mg QE (quercetin equivalent)/g of dry extract.

### 2.6. Antioxidant Activities Evaluation

#### 2.6.1. DPPH Radical Scavenging Assay

The DPPH (1,1-diphenyl-2-picrylhydrazyl) radical scavenging capacities of the extracts and compounds were determined through the Brand Williams technique [29], with minor modification, using gallic acid and quercetin as the positive controls. A total of 1 mL of DPPH 6 × 10^−5^ M solution was mixed with 33 μL of methanolic solution of each extract and compounds in various concentrations of 159.73, 79.87, 39.93, 19.97, 9.98, 4.99, 2.50, and 1.25 µg/mL. The samples were then incubated for 20 min at 37 °C in a water bath and subjected to UV-Vis spectrophotometric analysis at 517 nm (As). Meanwhile, a mixture of 33 μL of methanol and DPPH solution was used as the blank sample (Ab). Subsequently, the DPPH inhibitory activity was calculated using the equation [(Ab − As)/Ab] × 100%.

#### 2.6.2. ABTS Radical Scavenging Assay

The antioxidant activity was also evaluated with free radical ABTS (2,2″-azinobis (3-ethyl benzothiazoline-6-sulfonic acid) using the reported method [30]. For this evaluation, 5.0 mL of 7 mM ABTS stock solution were mixed with 88 μL of 140 mM potassium peroxydisulfate (K_2_S_2_O_6_) and stored in the dark at room temperature for 12–16 h before use. The mixture was then diluted in 99.5% ethanol to obtain a working solution with an absorbance of 0.7 (±0.02) at 734 nm. Subsequently, 1 mL of working solution and 10 μL of each sample in various concentrations (49.51, 24.75, 12.38, 6.19, 3.09, 1.55, and 0.78 µg/mL) were manually shaken for 10 s then incubated at 30 °C for 4 min. The absorbance of each solution was then measured at 734 nm with a UV-Vis spectrophotometer (As), and ethanol was used as a blank sample. Finally, the ABTS inhibitory activity was calculated with the equation [(Ab − As)/Ab] × 100%. Additionally, quercetin and gallic acid were used as positive controls.

#### 2.6.3. FRAP Assay

The ferric-reducing antioxidant power (FRAP) assay was determined using the reported method [31] with slight modification. The reduction of colorless ferric complex (Fe^3+^-tripyridyltriazine) to strongly blue-colored ferrous complex (Fe^2+^-tripyridyltriazine) is often evaluated by measuring the change of absorbance at 593 nm. For this analysis, the working FRAP reagent was prepared by mixing 300 mM acetate buffer pH 3.6, 10 mM 2,4,6-Tris(2-pyridyl)-s-triazine (TPTZ) (Sigma-Aldrich) solution in 40 mM HCl, and 20 mM FeCl_3_·6H_2_O in the ratio of 10:1:1, while the standard curve of FRAP was prepared using FeSO_4_·7H_2_O. About 100 μL of sample solution, 900 μL of distilled water, and 2 mL of FRAP reagent were mixed and incubated for 30 min in a dark room at 37 °C. The mixture’s absorbance was then measured at 593 nm, using a mixture of 2 mL of FRAP reagent and 1 mL of H_2_O as the blank sample. Subsequently, the FRAP values were stated as mM Fe^2+^/g of sample ((FRAP value of sample (μM) = abs (sample) × FRAP value of std (μM)/abs (std)).

### 2.7. In Vitro Cytotoxicity Assay

The in vitro cytotoxic assay against the HeLa (human cervix adenocarcinoma), MCF-7 (human breast adenocarcinoma), A549 (human lung carcinoma), and B16 (murine melanoma) cancer cell lines was performed using the PrestoBlue (PB) Assay with minor modification [32]. For this assay, the cells were preserved with a complete RPMI medium containing 10% (*v/v*) FBS and 50 μL/50 mL antibiotics. Cell cultures at a density of 170,000 cells/mL medium were then incubated in a 96 well plate at 37 °C in 5% CO_2_ for 24 h. Subsequently, the medium was removed and a fresh medium containing the sample in concentration variations of 300.00, 150.00, 75.00, 37.50, 18.75, 9.38, 4.69, and 2.34 µg/mL were added, and the plate was incubated for 48 h. This was followed by the addition of PrestoBlue reagent (Thermo Fisher Scientific, Uppsala, Sweden) and measurement of the mixture’s absorbance at 570 nm using a multimode reader. The IC_50_ values were then decided by the linear regression method, using Cisplatin as the positive control.

### 2.8. In Vitro Antiplasmodial Assay

The in vitro antiplasmodial evaluation against chloroquine-sensitive *P. falciparum* strain 3D7 was performed using the *lactate dehydrogenase* (LDH) method with slight modification [33], with chloroquine as the positive control. Furthermore, the samples were made using the serial dilution formula (50, 10, 5, 1, 0.5, 0.1, 0.05, and 0.01 μg/mL), while the plasmodial parasites used were the ring stage with 0.3% parasitemia and 2% hematocrit. For the assay, 1 μL of the sample solutions of various concentrations was poured into a well plate and 99 μL of parasites were added. The well plate was then incubated in a chamber with mixed atmospheric gas (5% O_2_, 5% CO_2_, and 90% N_2_) at 37 °C for 72 h, harvested, and stored at −30 °C for 24 h. Subsequently, the parasite growth was determined through an LDH assay performed by reading the absorbance at 650 nm with a SpectraMax Paradigm^®^ Multi-Mode microplate reader.

### 2.9. In Silico Molecular Docking Study and ADMET Prediction

The in silico study was performed using the Toshiba Portege Computer tool, Intel^®^ Core ™ i7-6600U CPU @ 2.60GHZ 2.80GHZ, 8.00 GB RAM, Intel HD Graphics 520, while the molecular docking was studied using Molegro Virtual Docker 5.5. Additionally, the ligands’ structures were drawn using ChemDraw 2018, minimized by Chem3D 2018, and saved as .mol2 format files.

The 2D structures of compounds 1–3 were drawn using ChemDraw 18.0 (PerkinElmer, Waltham, MA, USA) then converted into 3D structures. Subsequently, the compound’s minimum energy was calculated using Chem 3D 18.0 (PerkinElmer, Waltham, MA, USA) and stored as the mol2 {SYBYL2 (*. Mol2)} format. Meanwhile, the crystal structure of alpha-estrogen receptor (PDB ID: 3ERT) with the ligand 4-hydroxytamoxifen and *P. falciparum* lactate dehydrogenase receptor (PDB ID: 1CET) with chloroquine ligand were obtained from the Protein Data Bank. The docking results were expressed as MolDock Score (MDS), the minimum energy required in the process of ligand–receptor interaction. In addition, the absorption, distribution, metabolism, excretion, and toxicity (ADMET) properties of the most active compound were calculated using pkCSM (http://biosig.unimelb.edu.au/pkcsm, accessed on 22 June 2021).

## 3. Results and Discussion

### 3.1. Identification of the Compounds

**Figure 2:**
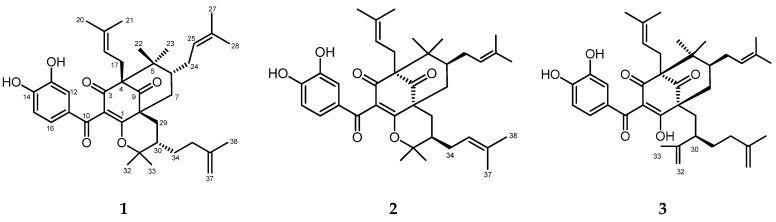
The structures of the polyprenylated benzophenones (compounds **1**–**3**) isolated from the root bark of *G. celebica*.

All isolated compounds were elucidated by spectroscopic methods including IR, as well as NMR analysis, and were compared with literature data. A total of three polyprenylated benzophenones: (-)-cycloxanthochymol (**1**) [34], isoxanthochymol (**2**), and xanthochymol (**3**) [35], were obtained from the phytochemical investigation of EtOAc extract of the root bark of *G. celebica* (Figure 2). Based on the literature review, there are no known reports on polyprenylated benzophenones isolated from *G. celebica*.

#### 3.1.1. (-)-Cycloxanthochymol (**1**)

White powder; mp: 222–224 °C; UV (MeOH) λ_max_: 278 and 224 nm; IR v_max_ (KBr): 3468, 2974, 2930, 1720, 1678, 1601, and 1298 cm^−1^; for ^1^H (500 MHz, CD_3_OD) and ^13^C NMR (125 MHz, CD_3_OD) spectroscopic data (Table 1).

#### 3.1.2. Isoxanthochymol (**2**)

White powder; mp: 168–170 °C, UV (MeOH) λ_max_: 250, 276, and 344 nm; IR *v_max_* (KBr): 3448, 2976, 2921, 1726, 1657, 1599, and 1292 cm^−1^; for ^1^H (500 MHz, CD_3_OD) and ^13^C NMR (125 MHz, CD_3_OD) spectroscopic data (Table 1).

#### 3.1.3. Xanthochymol (**3**)

Yellow needle; mp: 144–146 °C, UV (MeOH) λ_max_: 256 and 338 nm; IR v_max_ (KBr): 3304, 2936, 2961, 1726, 1632, 1611, and 1298 cm^−1^; for ^1^H (500 MHz, CD_3_OD) and ^13^C NMR (125 MHz, CD_3_OD) spectroscopic data (Table 1).

The ^1^H NMR data of compound **1** showed nine methyl signals at δ_H_ 0.84–1.73, six methylenes at δ_H_ 1.52–3.09, two methines at δ_H_ 1.33 (1H, m) and 1.52 (1H, m), two terminal olefinic protons at δ_H_ 4.79 (2H, s), and two olefinic protons indicating prenyl groups at δ_H_ 4.91 (1H, overlap) and 4.92 (1H, overlap). Furthermore, the proton signals for the 1,3,4-trisubstituted benzene ring at δ_H_ 6.70 (1H, d, J = 8.0), 6.95 (1H, dd, J = 8.0, 2.1), and 7.28 (1H, d, J = 2.1) represented the structure containing the AMX spin coupling. Due to overlapping ^1^H signals, DEPT-135 and HSQC were used to confirm the number and type of prenyl groups. The ^13^C NMR and DEPT-135 showed a methylene carbon at δ_C_ 39.7; a methine at δ_C_ 47.4; three sp^3^ quaternary carbon at δ_C_ 47.1, 52.9, and 69.5; an isolated carbonyl carbon at δ_C_ 207.9; a conjugated carbonyl carbon at δ_C_ 194.2; as well as an enolized 1,3-diketo group at δ_C_ 173.7, 126.8, and 196.4. These characteristic peaks were assigned from the combination of ^13^C NMR (Table 1) and HSQC data, indicating the bicyclo[3.3.1]nonane skeleton in the benzophenone derivative [36]. After comparison with published literature data, compound **1** was identified as (-)-cycloxanthochymol.

Compounds **1** and **2** were discovered to possess similar ^1^H NMR and ^13^C NMR data. However, the significant difference was the change of a dimethylallyl group to a prenyl group. The ^1^H NMR of compound **2** showed 10 methyl signals at δ_H_ 0.77–1.79, five methylenes at δ_H_ 1.04–3.03, and three olefinic protons indicating a prenyls group at δ_H_ 4.86 (1H, overlap), 5.16 (1H, t), and 5.21 (1H, t). After comparison with published literature data, compound **2** was identified as isoxanthochymol.

A comparison of the ^1^H NMR and ^13^C NMR data of compounds **3** and **1** showed a major structural difference at the opening of the pyran ring. The ^1^H NMR of compound **3** showed signals for eight methyls at δ_H_ 1.00–1.74, and four-terminal olefinic protons at δ_H_ 4.50 (2H, brs) and 4.64 (2H, d, J = 7.3). However, the carbon signals of the enolized 1,3-diketo group (C-1 and C-3) and carbon signals of the chiral center C-4 and C-8 disappeared due to the tautomerization keto-enol effect. After comparison with published literature data, compound **3** was identified as xanthochymol.

### 3.2. Total Phenolic and Flavonoid Contents of the G. celebica Extracts

Table 2 shows the TPC and TFC values of four different extracts from the root bark of *G. celebica*. The TPC values of the *G. celebica* extracts were determined from a linear gallic acid standard curve ($y=0.0042x+0.0234,\;R^{2}=0.9901$) and was discovered to be the highest in the *n*-hexane extract, followed by the EtOAc and CH_2_Cl_2_ extracts. This implies the phenolic compounds in the root bark of *G. celebica* were mostly non-polar and semi-polar. This fact was supported by Ramirez et al., who reported the high TPC value of *n*-hexane extract of epicarp and leaves of *G. madruno* [37]. Additionally, Menon et al. proved that some xanthones were isolated from the *n*-hexane extract of *G. wightii* [38]. Meanwhile, the TFCs of the *G. celebica* extracts were determined from a linear quercetin standard curve ($y=0.0337x,\;R^{2}=0.9973$). The results showed the flavonoid compounds contained in *G. celebica* were mostly non-polar to semi-polar. Based on the comparison of the TFC values (27.11 ± 0.24 to 35.73 ± 0.13 mg QE/g extract) and the TPC counterpart (112.84 ± 1.89 to 188.00 ± 1.78 mg GAE/g extract), flavonoids were not the major phenolic compound present in the *G. celebica* root bark. Therefore, other phenolic group compounds, including xanthones, polyisoprenylated benzophenones, biphenyls, and depsidones, possibly contribute to the extracts’ high TPC value.

### 3.3. Antioxidant Activity

The antioxidant potential assay of the extracts and isolated compounds from *G. celebica* based on a single electron transfer reaction or hydrogen atom transfer reaction comprised the 2,2-diphenyl-1-picrylhydrazyl (DPPH) radical scavenging activity, 2,2′-azino-bis (3-ethylbenzothiazoline-6-sulfonic acid) (ABTS) radical scavenging activity, and ferric-reducing antioxidant power (FRAP). Table 3 shows the antioxidant activities of the extracts and compounds, with the highest antioxidant activity exhibited by the CH_2_Cl_2_ extract for DPPH (IC_50_ 7.45 ± 0.04 μg/mL), followed by the n-hexane extract for ABTS assay (IC_50_ 5.96 ± 0.00 μg/mL), and the EtOAc extract by FRAP assay (66.36 ± 5.05 μM Fe^2+^/g). Furthermore, xanthochymol **3** exhibited stronger antioxidant activities by DPPH assay (2.33 ± 0.06 μg/mL or 3.87 ± 0.10 μM), ABTS assay (0.53 ± 0.01 μg/mL or 0.88 ± 0.02 μM), and FRAP assay (44.28 ± 0.79 μM Fe^2+^/g). In this study, the antioxidant activity of xanthochymol **3** by DPPH assay was higher, compared to the data reported by [39], with IC_50_ of 53 ± 1.0 μM.

The ABTS result provides a better description of the antioxidant capacity compared to the DPPH assay, presumably due to the different reactions of phenolic compounds and free radicals in the aqueous phase (ABTS assay system) and organic phase (DPPH assay system) [40,41]. In addition, the ABTS assay is applicable for both lipophilic and hydrophilic systems, while the DPPH assay is only relevant to the hydrophobic system [38].

The FRAP evaluation indicates a sample’s capacity to participate in a one-electron redox reaction [42]. According to Table 3, all the extracts exhibited higher FRAP values, compared to ascorbic acid as the positive control, particularly the EtOAc extract, which exhibited a two-fold higher value. The results indicate the antioxidant potential of *G. celebica* extracts against the oxidative effects of reactive oxygen species [43]. Furthermore, the extracts’ antioxidant power was proportional to the TPC and TFC values, and this is probably due to the presence of flavonoids and other phenolic compounds, which tend to act as electron donors, consequently neutralizing free radicals. Additionally, the antioxidant power determined by FRAP exhibited a linear relationship with the radical scavenging activity of the polyprenylated benzophenones determined by DPPH and ABTS. Therefore, an increase in the FRAP value implies an increase in the compounds’ ability to scavenge free radicals. Meanwhile, the reducing power of the benzophenones is possibly due to the presence of hydroxyl groups, which act as electron donors to the reactive oxygen species [26,44].

### 3.4. In Vitro Cytotoxic Activity

Based on previous reports, several polyprenylated benzophenones from the genus Garcinia exhibit potent biological activities, especially cytotoxicity against cancer cell lines [14,20,45]. Numerous reports have explained the ability of polyprenylated benzophenones to inhibit various cancer cells’ growth through various mechanisms of action, including apoptosis, cell cycle arrest, and endoplasmic reticulum response [20,46,47,48,49]. For instance, xanthochymol, garcinol, and isogarcinol inhibit human leukemia cell lines’ growth due to apoptosis mediated by the activation of caspase-3 [46]. Meanwhile, other polyprenylated benzophenones, including isogarcinol, isoxanthochymol, and guttiferone E, strongly induced apoptosis in the leukemia cell line CCRF-CEM through activation of caspases 3/7, caspase 8, and caspase 9 [48]. Furthermore, xanthochymol, guttiferone E, and guttiferone H induced G1 arrest of the cell cycle and terminal caspase activation associated with interference of the mitochondrial membrane potential and subsequent activation of the endoplasmic reticulum stress [19].

Based on the cytotoxicities above, the cytotoxicities of compound **1** to **3** were evaluated against 4 cancer cell lines (HeLa, MCF-7, A549, and B16). According to the literature review, there are no known reports on the cytotoxicity of the isolated benzophenones against the B16 cancer cell line. Table 4 shows compounds **1** to **3** exhibited adequate cytotoxicity, with IC_50_ values of 10.18 to 16.94 μM, compared to cisplatin as the positive control. Compound **2** exhibited the highest cytotoxicity against the HeLa, MCF-7, and B16 cell lines, with IC_50_ values of 10.18, 10.73, and 12.39 μM, respectively. However, compound **1** exhibited the highest cytotoxicity against A549, with an IC_50_ value of 13.77 μM. In addition, the structure–activity relationship (SAR) analysis showed compounds **1** and **2** were more active, compared to compound **3**. Therefore, the opening of the pyran cycle of C-31 reduces the cytotoxicity activity [48]. Additionally, the change of a prenyl group to a dimethyl allyl group at C-30 possibly reduces the compound’s cytotoxicity [50].

### 3.5. In Vitro Antiplasmodial Activity

The in vitro antiplasmodial activity of compounds **1** to **3** was assessed against the chloroquine-sensitive *P. falciparum* strain 3D7. Table 5 shows compound **2** was active against *P. falciparum* strain 3D7 with an inhibition percentage of 83.97 ± 0.47% (IC_50_ 2.99 ± 0.20 μM) at 10 μg/mL. This is the first known report of compound **2** isolated from Garcinia genera having antiplasmodial activity against the 3D7 strain. Previous studies have reported the antiplasmodial activity of compound **2** against NF54, Ghana, D6, and W2 strains [22,24,51].

Based on the discussion above, the phenolic compounds of polyprenylated benzophenones have interesting biological activity. Xanthochymol has the best antioxidant activity while isoxanthochymol has the best cytotoxicity and antiplasmodial activities. The high xanthochymol activity was influenced by the higher number of hydroxyl groups [52]. Furthermore, the high activity of isoxanthochymol is probably influenced by the formation of the pyran ring and the presence of a prenyls group substitute in the benzophenone skeleton [22,47,50].

### 3.6. Molecular Docking Studies

#### 3.6.1. Human Alpha-Estrogen Receptor

Estrogen, the essential female sex hormone, had been reported to play a significant role in the progression of breast cancer. Furthermore, the estrogen receptor mediates the effect of estrogen, with about 70% of all breast cancers expressing the estrogen receptor alpha [53,54,55]. In this study, the molecular docking of isolated polyprenylated benzophenones **1** to **3**, compared to 4-hydroxytamoxifen, was studied toward human alpha-estrogen receptor (PDB ID: 3ERT) using Molegro Virtual Docker 5.5. Based on the interaction analysis, 4-hydroxytamoxifen associates through hydrogen bonding with the residues Arg394 and Glu353, steric interaction at residue site of Ala350, and electrostatic interaction at residue site Asp351.

**Figure 3:**
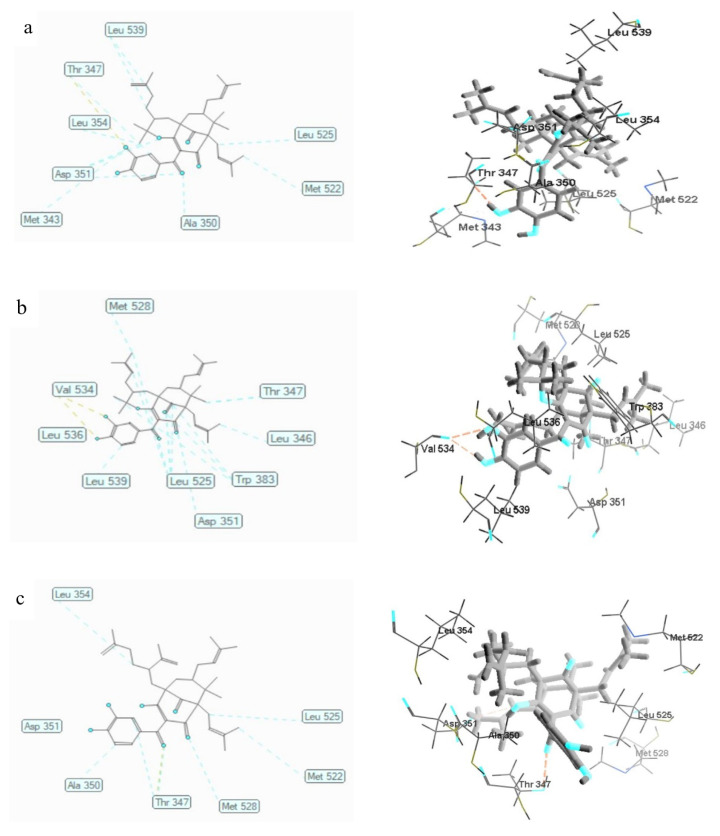
The 2D and 3D interaction of **1** (**a**), **2** (**b**), and **3** (**c**) with human alpha-estrogen receptor (3ERT). Hydrogen bonds are shown as yellow dotted lines while steric interactions are depicted as blue dotted lines. Carbon atoms are represented in gray, oxygen in blue, and hydrogen in black.

The docking results showed the MDS of compounds **1**, **2**, and **3** were −144.638 ± 1.41, −139.590 ± 0.37, and −162.832 ± 2.24 kcal/mol, respectively (Table 6). Compound **3** possessed the least bonding energy with MDS −162,832 ± 2238 kcal/mol, and was, therefore, predicted to have the most stable molecular binding with an alpha-estrogen receptor. Table 6 and Figure 3 show the binding mode of compounds **1** to **3**, where the three compounds were discovered to inhibit the alpha-estrogen receptor through hydrogen bonding and steric interactions. These compounds are bound to the active site of 3ERT through hydrogen bonding interaction of free hydroxyl groups and carbonyl with Thr347 and Val534 (2) residues. In addition, the steric interactions of compound **1** were formed by the oxygen atoms of hydroxyl and carbonyl groups with Met343, Ala350, and Asp351 residues; the benzophenones backbone and pyran ring with Thr347, Asp351 (2), and Leu354; and prenyl and dimethylallyl groups with Leu525, Met522, and Leu539 (2). Similar interactions were also formed by compound **2** with Leu525 (4), Trp383 (4), Thr347, Leu346, Asp351, Leu539, Leu536, as well as Met528, and by compound **3** with Thr347 (2), Ala350, Asp351, Leu354, Leu525, Met522, as well as Met528.

#### 3.6.2. *Plasmodium falciparum* Lactate Dehydrogenase Enzyme

**Figure 4:**
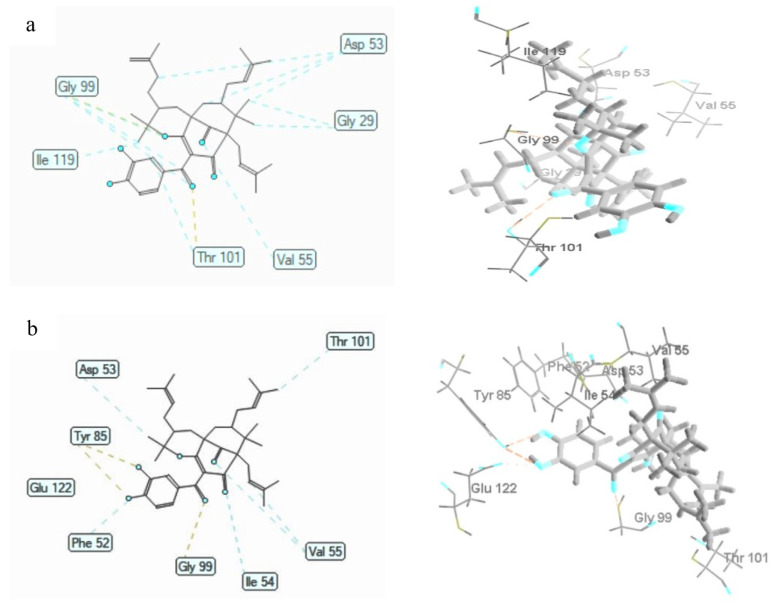

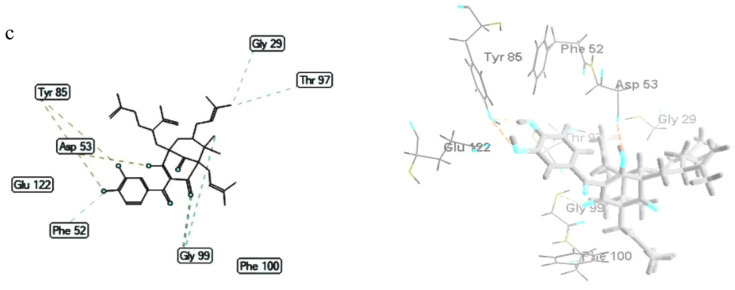
The 2D and 3D interaction of **1** (**a**), **2** (**b**), and **3** (**c**) with Plasmodium falciparum lactate dehydrogenase enzyme (1CET). Hydrogen bonds are shown as yellow dotted lines while steric interactions are depicted as blue dotted lines. Carbon atoms are represented in gray, oxygen in blue, and hydrogen in black.

The molecular docking of polyprenylated benzophenones **1** to **3** was also studied on *P. falciparum* lactate dehydrogenase enzyme (PDB ID: 1CET). The MDS of compounds **1** to **3** were discovered to be −154.72 ± 0.48, −147.43 ± 1.16, and −145.56 ± 0.84 kcal/mol, respectively (Table 7). Compound **1** possessed the least bonding energy with an MSD value of −154.72 ± 0.48 kcal/mol, and therefore, exhibited the most stable molecular binding with the *P. falciparum* lactate dehydrogenase receptor. The polyprenylated benzophenones **1** to **3** bound to 1CET through hydrogen bonding of the oxygen atom of the carbonyl group (C10) with Thr101 and Gly99 residues, the oxygen atom of hydroxyl groups (C13 and C14) with Tyr85 residue, the oxygen atom of the hydroxyl group (C1) with Asp53, and the oxygen atom of the pyran ring with Gly99 (Table 7 and Figure 4). Furthermore, compound **1** exhibited steric interactions of the prenyl groups with Asp53 (2) residue, and the benzophenone skeleton with Asp53, Gly29 (2), Gly99 (2), Val55, Thr101, and Ile119 residues. Meanwhile, the steric interactions of compound **2** were established by the oxygen atom of the carbonyl group (C3) with Ile54 residue and the hydroxyl group with Phe52 residue, prenyl groups with Thr101 and Val55 residues, and the benzophenone skeleton with Val55 and Asp53 residues. The Phe52 and Gly99 residues of 1CET were bound to hydroxyl and carbonyl groups, while the Gly29 and Thr97 residues were bound to the prenyl group, and the Gly99 residue was bound to the benzophenone skeleton of compound **3** by steric interactions.

#### 3.6.3. ADMET Profiles

According to the in silico study of the absorption, distribution, metabolism, excretion, and toxicity properties of isolated compounds **1** to **3**, as derived from the pkCSM online tool, all compounds had a molecular weight of 602.812 (>500) g/mol. The Caco-2 permeability for the prediction of orally administered drug of compounds **1** to **3** was above 0.90 (Table 8), indicating all compounds possessed high permeability. Furthermore, all benzophenone compounds had high intestinal absorption, and are, therefore, well absorbed in the small intestine. Higher human intestinal absorption implies the compounds tend to be better absorbed within the gastrointestinal tract after oral administration [56]. Additionally, the transdermal drug efficacy illustrated by the compounds’ skin permeability, ranged from −2.75 to −2.73 cm/h (<−2.5). Consequently, compounds **1** to **3** are expected to penetrate the skin properly [57].

Compounds **1** to **3** also possess log VDss values below −0.15, indicating the compounds disperse in blood plasma. The ability of a drug to permeate the brain is indicated by the blood–brain barrier (BBB) permeability. Compounds are able to permeate the blood–brain barrier in cases where the logBB is above 0.3, while compounds with logBB below −1 are hardly dispersed to the brain. In this study, compounds **1** and **3** were discovered to have logBB values between −1 and 0.3 while compound **2** had a logBB value below −1. This indicates that compounds **1** and **3** are somewhat able to permeate the blood–brain barrier but compound **2** is not [57].

Table 8 shows compounds **1** to **3** were non inhibitors for CYP2D6 and CYP3A4. Generally, the compounds do not interfere with the cytochrome CYP450 biotransformation. The cytochrome P450 is a crucial detoxification enzyme in the human body.

Drug excretion from the human body is represented by total clearance and renal OCT2 substrate. The drug clearance is determined by the log (CLtot) of compound (ml/min/kg), from a combination of hepatic clearance and renal clearance. In this study, the total clearance of compounds **1** to **3** ranged from −0.02 to 0.29, and this is categorized as low clearance (Log CLtot < 2) [58]. Therefore, the three compounds are expected to be discharged slowly. Additionally, the adverse interactions of compounds **1** to **3** with OCT2 inhibitors as denoted by the OCT2 substrate parameter showed that the three compounds exhibit no potential contraindication.

The toxicity of promising medicinal compounds is a highly crucial factor. In this study, the acute toxicity (LD_50_) of compounds **1** to **3** ranged from 3.36 to 3.80 mol/kg, while the chronic toxicity of the compounds (LOAEL) ranged from 1.72 to 1.75 (52.48 to 56.23 mg/kg bw/day). Compound **2** was discovered to be the most toxic of the three benzophenones. The hepatotoxicity descriptor declared that compounds **1** to **3** were not hepatotoxic.

## 4. Conclusions

This study performed novel extractions of three known polyprenylated benzophenones from the root bark of *G. celebica*. The compounds were isolated from the EtOAc extract, and structural elucidation was carried out through spectroscopic analysis and literature data comparison. According to the results, the highest antioxidant activities were recorded for the CH_2_Cl_2_ extract by DPPH assay (7.45 ± 0.04 μg/mL), followed by the *n*-hexane extract by ABTS assay (5.96 ± 0.00 μg/mL), and EtOAc extract by FRAP assay. Compound **3** exhibited the highest antioxidant activity by DPPH, ABTS, and FRAP assay, with an IC_50_ value of 3.87 ± 0.10 μM, 0.88 ± 0.02 μM, and 44.28 ± 0.79 μM Fe^2+^/g, respectively. Meanwhile, compound **2** exhibited the strongest cytotoxicity against HeLa, MCF-7, and B16 cell lines, with IC_50_ values of 10.18, 10.73, and 12.39 μM, respectively, and was also active against *P. falciparum* strain 3D7, with an IC_50_ value of 2.99 ± 0.20 μM at 10 μg/mL. Based on the molecular docking result, compound **3** possessed the most stable molecular binding with an alpha-estrogen receptor (MDS −162,832 ± 2238 kcal/mol) and compound **1** with *P. falciparum* lactate dehydrogenase receptor (MDS −154.72 ± 0.48 kcal/mol). Additionally, the ADMET prediction indicated compounds **1**–**3** were potential oral drug candidates. These findings imply the value of *G. celebica* as a natural resource in the medicinal area of malaria and cancer.

## Figures and Tables

**Table 1:** ^1^H (500 MHz) and ^13^C (125 MHz) NMR spectroscopic data of Compounds **1**–**3** (in ppm).

Position	1 (CD_3_OD)		2 (CD_3_OD)		3 (CD_3_OD)	
	δ_C_	δ_H_ (J in Hz)	δ_C_	δ_H_ (J in Hz)	δ_C_	δ_H_ (J in Hz)
1	173.7		172.9		195.8 *^b^*	
2	126.8		128.0		117.9	
3	196.4		196.0		- *^c^*	
4	69.5		71.7		- *^c^*	
5	47.1		46.9		50.2	
6	47.4	1.52, m	42.4	1.98, m	47.9	1.50 *^a^*
7	39.7	2.27, m	43.3	2.17, dd (14.1, 4.5)	43.7	2.05, m
		2.04, m		1.56, t (13.0)		2.05, m
8	52.9		54.2		- *^c^*	
9	207.9		207.2		210.6	
10	194.2		195.1		195.8	
11	131.1		130.7		129.5	
12	115.9	7.28, d (2.1)	116.3	7.29, d (2.1)	117.3	7.19, d (2.2)
13	146.7		146.7		146.3	
14	152.6		152.8		152.5	
15	115.4	6.70, d (8.0)	115.6	6.75, d (8.3)	115.1	6.71, d (8.3)
16	124.6	6.95, dd (8.0, 2.1)	124.8	7.06, dd (8.2, 2.1)	125.2	6.98, dd (8.3, 2.2)
17	26.6	2.46, dd (13.5, 6.0)	25.8	2.44, dd (13.8, 5.8)	27.0	2.71, dd (13.30, 9.45)
		2.64, dd (14.0, 6.0)		2.63, dd (13.6, 8.0)		2.57, m
18	121.2	4.91 *^a^*	121.4	4.86 *^a^*	121.4	5.05, m
19	135.5		135.1		135.8	
20	26.6	1.59, s	26.4	1.61, s	26.5	1.74, s
21	18.3	1.59, s	18.3	1.60, s	18.3	1.69, s
22	22.9	1.15, s	22.6	1.09, s	23.2	1.15, s
23	27.1	0.99, s	16.3	0.77, s	27.3	1.00, s
24	30.5	2.15, m	30.6	1.85, m	30.3	2.05, m
		2.67, m		2.07, m		2.05, m
25	126.3	4.92 *^a^*	122.9	5.21, t	125.6	4.90 *^a^*
26	134.0		134.7		133.6	
27	26.1	1.69, s	26.0	1.79, s	26.0	1.66, s
28	18.6	1.67, s	18.1	1.65, s	18.2	1.50, s
29	28.8	1.07, br d (13.5)	28.5	1.04, t (13.6)	37.7	1.90, m
		3.09, dd (14.0, 3.4)		3.03, dd (14.2, 3.65)		1.90, m
30	42.9	1.33, m	44.6	1.40, tt (13.5)	44.7	2.57, m
31	88.5		88.5		148.9	
32	28.8	0.84, s	21.5	1.24, s	113.6	4.50, br s
33	21.5	1.24, s	29.0	0.98, s	17.8	1.59, s
34	29.3	1.15 *^a^*	28.7	1.79 *^a^*	32.7	1.50 *^a^*
		1.52, m		2.23, d (14.5)		1.42, m
35	36.2	2.15, m	123.9	5.16, t	36.8	1.83, m
		2.27, m				1.83, m
36	146.0		134.2		146.9	
37	111.9	4.79, s	26.0	1.73, s	110.5	4.64, d (7.3)
38	22.3	1.73, s	18.1	1.62, s	22.8	1.69, s

*^a^* Overlapping signals; *^b^* Data was extracted from the HMBC spectrum; *^c^* Not detected; s: singlet; d: doublet; t: triplet; m: multiplet; dd: doublet of doublet; br: broad; tt: triplet of triplet.

**Table 2:** The TPC and TFC values of *G. celebica* root bark extracts.

Extract	TPC (mg GAE/g Extract)	TFC (mg QE/g Extract)
*n*-Hexane	188.00 ± 1.78	33.07 ± 0.76
CH_2_Cl_2_	164.35 ± 1.19	31.03 ± 2.28
EtOAc	180.06 ± 1.07	35.73 ± 0.13
MeOH	112.84 ± 1.89	27.11 ± 0.24

Data are expressed as mean ± SD of triplicate experiments.

**Table 3:** The antioxidant activities of *G. celebica* root bark extracts and isolated compounds **1**–**3**.

Extracts/Compounds	Antioxidant Capacity				
	DPPH		ABTS		FRAP (μM Fe^2+^/g)
	IC_50_ (μg/mL)	IC_50_ (μM)	IC_50_ (μg/mL)	IC_50_ (μM)	
*n*-Hexane	7.86 ± 0.07	-	5.96 ± 0.00	-	35.64 ± 1.56
CH_2_Cl_2_	7.45 ± 0.04	-	6.43 ± 0.02	-	34.58 ± 1.67
EtOAc	7.55 ± 0.06	-	6.01 ± 0.01	-	66.36 ± 5.05
MeOH	11.40 ± 0.00	-	7.63 ± 0.01	-	38.75 ± 2.41
**1**	3.36 ± 0.03	5.58 ± 0.05	0.78 ± 0.02	1.30 ± 0.04	22.61 ± 0.06
**2**	2.98 ± 0.05	4.95 ± 0.08	0.70 ± 0.02	1.16 ± 0.04	24.58 ± 0.29
**3**	2.33 ± 0.06	3.87 ± 0.10	0.53 ± 0.01	0.88 ± 0.02	44.28 ± 0.79
Gallic acid	0.55 ± 0.00	3.22 ± 0.02	0.12 ± 0.00	0.68 ± 0.00	Nt
Quercetin	1.34 ± 0.02	4.43 ± 0.06	0.06 ± 0.00	0.19 ± 0.00	Nt
Ascorbic acid	Nt		Nt		30.62 ± 0.27

Nt: not tested.

**Table 4:** The cytotoxic activity of the compounds after 72 h of treatment.

Compounds	IC_50_ (μM)			
	HeLa	MCF-7	A549	B16
**1**	13.77 ± 0.07	13.54 ± 0.01	13.77 ± 0.20	12.46 ± 0.08
**2**	10.18 ± 0.51	10.73 ± 0.14	13.99 ± 0.09	12.39 ± 0.11
**3**	15.48 ± 0.13	14.93 ± 0.52	16.10 + 0.18	16.94 ± 0.15
Cisplatin	19	53	18	43

**Table 5:** Antiplasmodial activity against the chloroquine-sensitive *P. falciparum* strain 3D7.

Compound	% Inhibition ± SD (10 μg/mL)	IC_50_ ± SD (μM)
**1**	<10	Nt
**2**	83.97 ± 0.47	2.99 ± 0.20
**3**	<10	Nt
chloroquine	98.8 ± 0.25	0.006 ± 0.01

% Inhibition <10% = inactive, Nt = Not tested.

**Table 6:** MolDock score and interacting residues of **1**, **2**, and **3** to human alpha-estrogen receptor (3ERT).

Ligands	MolDock Score (kcal/mol)	Hydrogen Bonding	Steric Interaction	Electrostatic Interaction
**1**	−144.638 ± 1.41	Thr347	Asp351 (3); Leu539 (2); Ala350; Met343; Leu354; Thr347; Leu525; Met522	
**2**	−139.590 ± 0.37	Val534 (2)	Leu525 (4); Trp383 (4); Thr347; Leu346; Asp351; Leu539; Leu536; Met528	
**3**	−162.832 ± 2.24	Thr347	Thr347 (2); Ala350; Asp351; Leu354; Leu525; Met522; Met528	
4-hydroxtamoxifen	−165.042 ± 0,48	Arg394; Glu353	Ala350	Asp351

**Table 7:** MolDock score and interacting residues of **1**, **2**, and **3** to *P. falciparum* lactate dehydrogenase enzyme (1CET).

Ligands	MolDock Score (kcal/mol)	Hydrogen Bonding	Steric Interaction
**1**	−154.72 ± 0.48	Gly99; Thr101	Gly29 (3); Gly99 (2); Asp53 (3); Val55; Ile119, Thr101
**2**	−147.43 ± 1.16	Tyr85 (2); Gly99; Glu122	Ile54 (3); Asp53; Thr101; Val55 (2), Phe52
**3**	−145.56 ± 0.84	Tyr85 (2); Gly99; Asp53	Gly29; Gly99 (2); Thr97; Phe52
Chloroquine	−108.99 ± 0.48	Gly29; Gly99; Ser28	Gly29; Gly99; Ala98; Asp53

**Table 8:** ADMET properties by the pkSCM online tool.

Ligands	Absorption			Distribution		Metabolism		Excretion		Toxicity		
	Caco-2 Permeability	Intestinal Absorption (Human)	Skin Permeability	VDss (Human)	BBB Permeability	CYP2D6 Inhibitor	CYP3A4 Inhibitor	Total Clearance	Renal OCT2 Substrate	Oral Rat Acute Toxicity (LD50)	Oral Rat Chronic Toxicity (LOAEL)	Hepatotoxicity
**1**	1.54	100.00	−2.74	−0.16	−0.51	No	No	0.04	No	3.36	1.74	No
**2**	1.26	92.65	−2.73	−0.46	−1.39	No	No	0.29	No	3.80	1.72	No
**3**	1.60	100.00	−2.75	−0.19	−0.48	No	No	−0.02	No	3.37	1.75	No
4−Hidroxytamoxifen	1.03	93.54	−2.74	0.31	−0.29	Yes	No	0.59	No	2.18	1.37	No
Chloroquine	1.259	89.44	−2.564	1.757	0.410	Yes	No	0.993	Yes	2.888	0.423	Yes

## Data Availability

Not applicable.

## Abbreviations

ABTS	2,2″-azinobis(3-ethyl benzothiazoline-6-sulfonic acid)
ADMET	Absorption Distribution Metabolism Excretion Toxicity
DPPH	1,1-diphenyl-2-picrylhydrazyl
FRAP	ferric reducing antioxidant power
PDB	protein data bank
pkCSM	small-molecule pharmacokinetics prediction
TPTZ	2,4,6-Tris(2-pyridyl)-s-triazine
